# Impact of certolizumab pegol on patient-reported outcomes in rheumatoid arthritis and correlation with clinical measures of disease activity

**DOI:** 10.1186/s13075-015-0849-1

**Published:** 2015-11-27

**Authors:** Janet Pope, Clifton O. Bingham, Roy M. Fleischmann, Maxime Dougados, Elena M. Massarotti, Jürgen Wollenhaupt, Benjamin Duncan, Geoffroy Coteur, Michael E. Weinblatt

**Affiliations:** St. Joseph’s Health Care, University of Western Ontario, London, ON Canada; Divisions of Rheumatology and Allergy, Johns Hopkins University, Baltimore, MD USA; Metroplex Clinical Research Center, University of Texas, Dallas, TX USA; Département de Rhumatologie, Paris Descartes University, 12 Rue de l’École de Médecine, 75006 Paris, France; Department of Medicine, Rheumatology, Immunology, Brigham and Women’s Hospital, Boston, MA USA; Klinik für Rheumatologie, Schön Klinik Hamburg Eilbek, Hamburg, Germany; UCB Pharma, Raleigh, NC USA; UCB Pharma, Brussels, Belgium

**Keywords:** Rheumatoid arthritis, Certolizumab pegol, TNF inhibitor, PROs, Biological therapy

## Abstract

**Introduction:**

The effect of certolizumab pegol (CZP) on patient-reported outcomes (PROs) was investigated in 1063 patients with rheumatoid arthritis (RA) from the REALISTIC trial (double-blind, placebo-controlled to week 12, open-label to week 28; randomized 4:1 [CZP:placebo]). Correlations between PROs and RA signs and symptoms, and the relative efficacy of these measures, were examined.

**Methods:**

Adults with RA and an inadequate response to at least one disease-modifying antirheumatic drug were enrolled. PROs assessed included physical function (using the Health Assessment Questionnaire-Disability Index), pain, fatigue, sleep disturbance, Patient Global Assessment of Disease Activity (PtGA), Routine Assessment of Patient Index Data 3 (RAPID3), and Rheumatoid Arthritis Disease Activity Index (RADAI).

**Results:**

Early significant and clinically meaningful improvements in all PROs were observed to week 12 with CZP vs. placebo and were maintained to the end of the trial (week 28). At week 12, up to one-third more CZP patients showed improvements compared with placebo that were greater than or equal to the minimal clinically important difference (MCID) in fatigue, sleep problems, pain, PtGA, RADAI, and RAPID3. The changes in PROs were correlated with clinical measures of disease activity, including the Disease Activity Score in 28 joints using C-reactive protein as well as tender and swollen joint counts.

**Conclusions:**

Rapid improvements in PROs were seen in patients with RA treated with CZP. The magnitude of improvement exceeded the MCID in multiple domains and demonstrated that CZP improves aspects of health-related quality of life that are meaningful to patients and superior to placebo. PROs provide information complementary to clinical outcomes in assessment of treatment benefits.

**Trial registration:**

ClinicalTrials.gov identifier: NCT00717236. Registered on 15 July 2008.

## Introduction

Patient-reported outcomes (PROs), such as physical function, pain, and fatigue, reflect the important effects of rheumatoid arthritis (RA) on the daily lives of people living with the disease. Several studies have suggested that PROs discriminate between treatment effects and physician-reported clinical outcomes [1–3]. By using both, a physician should have a more comprehensive assessment of a patient’s status. Assessment of PROs in clinical trials is now recommended by the U.S. Food and Drug Administration [4] and the European Medicines Agency [5] for the evaluation of medications for chronic diseases such as RA. There is interest in how PROs compare to each other, their sensitivity to change, and how they differentiate between treatments in blinded, randomized clinical trials (RCTs).

Self-reported disease activity questionnaires, such as the Rheumatoid Arthritis Disease Activity Index (RADAI) and the Routine Assessment of Patient Index Data 3 (RAPID3), offer a patient-focused approach to the clinical management of RA [6–8]. RAPID3 is an index of three PRO measures in the American College of Rheumatology (ACR) Core Data Set [9] (physical function, pain, and Patient Global Assessment of Disease Activity [PtGA]), but it does not include formal joint counts. RADAI includes a patient self-assessment of joint tenderness and pain in 16 joint areas, current and past global disease activity, and morning stiffness [6, 7]. Neither index employs a physician joint count or determination of an acute-phase reactant.

Most clinical trials have strict inclusion and exclusion criteria, excluding patients with many of the comorbidities commonly seen in clinical practice and enrolling a more homogeneous and less complicated population than that usually encountered in clinical practice settings. Evaluations of anti–tumor necrosis factor (anti-TNF) therapy in groups more representative of the clinical setting are lacking. The REALISTIC (RA EvALuation In Subjects receiving TNF Inhibitor Certolizumab pegol) study, which was double-blinded and placebo-controlled to week 12 (randomizing patients in a 4:1 fashion [certolizumab pegol:placebo]) and open-label thereafter, had fewer exclusion criteria than most clinical trials. This study demonstrated that the PEGylated Fc-free anti-TNF agent certolizumab pegol (CZP), either as monotherapy or in addition to current treatment, is efficacious on the basis of clinical response criteria in a broad group of patients with active, inadequately controlled RA [10].

In this article, we investigate the efficacy of CZP in patients with RA with regard to PROs of physical function, pain, fatigue, sleep, and PtGA in the REALISTIC study and examine the correlations between PROs and clinical indices of RA signs and symptoms, including the Disease Activity Score in 28 joints using C-reactive protein (DAS28[CRP]) and formal joint counts. Finally, the relative efficacy of composite PROs (RADAI and RAPID3) and clinical indices of RA signs and symptoms are compared to ascertain their respective sensitivity to therapeutic efficacy in the CZP-treated population.

## Material and methods

### Patients and study design

Detailed methods of the REALISTIC study have been published previously [10]. Briefly, the trial enrolled 1063 patients aged ≥18 years with adult-onset RA, as defined by the 1987 ACR criteria [11], of ≥3 months’ duration, with an unsatisfactory response to or intolerance of at least one disease-modifying antirheumatic drug (DMARD; methotrexate [MTX], leflunomide, sulfasalazine, chloroquine or hydroxychloroquine, azathioprine, and gold). Patients were stratified by baseline MTX use, prior anti-TNF use, and disease duration (<2 years vs. ≥2 years) and randomized 4:1 to receive, in addition to their existing treatment, either (1) a CZP 400-mg loading dose at weeks 0, 2, and 4, followed by CZP 200 mg every 2 weeks; or (2) placebo injection (control) every 2 weeks for the initial 12-week double-blind RCT. After completing the 12-week RCT, patients could enter the open-label phase and receive CZP 200 mg every 2 weeks (following the loading dose for patients originally randomized to placebo). The trial was carried out in compliance with the principles of the Declaration of Helsinki and was approved by the institutional review boards at each participating center. The full details of all institutional review boards are provided at the end of the main text in the Acknowledgments section. All patients provided written informed consent.

### Efficacy evaluations

PROs were secondary and exploratory endpoints in the REALISTIC study. They included assessments of physical function (Health Assessment Questionnaire-Disability Index [HAQ-DI]), arthritis pain, and PtGA. Pain and PtGA were both scored on 100-mm visual analogue scales (VASs) [12]. PtGA was scored from 0 (“very good, no symptoms”) to 100 (“very poor, severe symptoms”), and pain was scored from 0 (“no pain”) to 100 (“most severe pain”). The minimal clinically important difference (MCID) for both has been established as a 10-mm decrease (i.e., improvement) from baseline [13, 14]. The MCID for the HAQ-DI was prespecified as a 0.22-point improvement (i.e., −0.22 change), based on previous trials [15, 16].

RAPID3 is an index composed of three routine PROs assessed on a standard patient questionnaire [7]: the HAQ-DI, the pain VAS, and the PtGA VAS. It was investigated as a post-hoc analysis. To calculate the RAPID3 score, the raw 0–3 score for physical function in the HAQ-DI is multiplied by 3.33 and the pain and PtGA VAS scores are each divided by 10, to give scores of 0–10. The HAQ-DI, pain, and PtGA scores are then summed for a raw score of 0–30. The MCID for RAPID3 is a 3.6-point decrease from baseline [17].

Exploratory endpoints included assessments of fatigue according to the 10-point Fatigue Assessment Scale (FAS) [14], sleep quality and quantity as measured by the Sleep Problem Index II domain of the 12-item Medical Outcomes Study Sleep Scale (MOS-SPI) [18], and disease activity from the patient’s perspective as assessed with the RADAI [6].

Fatigue was scored from 0 (“no fatigue”) to 10 (“fatigue as bad as you can imagine”), with MCID defined as a 1-point decrease from baseline [14]. Sleep over the last 4 weeks was assessed using 12 questions relating to the time taken to fall asleep, quality of sleep, amount of waking during the night, and the effect of sleep on daytime functioning. The MCID reported for MOS-SPI is a decrease ≥6 [14].

The RADAI is a self-administered, 5-item questionnaire [6] used to assess the following: (1) global disease activity in the past 6 months, (2) disease activity in terms of tender and swollen joints, (3) arthritis pain, (4) duration of morning stiffness, and (5) tender joints (16-joint count). To calculate the RADAI joint score (RADAI-JS), each joint in the assessment of tender joints (item 5 above, 16-joint count) is scored from 0 to 3, and subsequently these scores are summed (maximal sum score 48) and multiplied by 10/48 to adjust the overall score to a range of 0–10. To calculate the RADAI total score (RADAI-TS), the five items are combined into a single index of patient-assessed disease activity with a range of 0–10. The MCID for the RADAI-TS is a 1-point decrease from baseline [19].

All PROs except MOS-SPI were assessed at weeks 0, 2, 6, 12, 20, and 28 and at completion of or withdrawal from the trial. MOS-SPI was assessed at all time points except week 2. We report the PRO findings for the 12-week, double-blind phase of the REALISTIC study and for the subsequent open-label phase through week 28.

### Statistical analysis

PRO analyses were carried out using the intention-to-treat (ITT) population, which included all randomized patients. The results are reported as ITT unless otherwise specified.

Least squares mean change from baseline (CFB) in HAQ-DI, pain, fatigue, sleep problems, PtGA, RAPID3, and RADAI-TS were obtained using analysis of covariance (ANCOVA) with factors for treatment, baseline MTX status, prior anti-TNF use, disease duration category (<2 years vs. ≥2 years), and baseline response as covariates. Missing data were accounted for by using the last observation carried forward. All reported *p* values and confidence intervals are nominal and can be interpreted only in an exploratory manner. *p* values were reported for odds ratios rather than for direct comparison between percentages of patients with improvements greater than or equal to the MCID.

Post hoc comparisons of the proportion of patients reporting improvements greater than or equal to the MCID for RADAI, RAPID3, and other PROs were performed. For exploratory purposes, logistic regression was performed with treatment, baseline MTX status, prior anti-TNF use, disease duration category (<2 years vs. ≥2 years), and baseline response as covariates.

Correlations between PROs and clinical disease activity measures of DAS28 using C-reactive protein (DAS28[CRP]), log(CRP), tender joint count (TJC), and swollen joint count (SJC) at week 12 were analyzed for all patients (ITT population) using Pearson correlations. They were interpreted as <0.3 = low correlation, ≥0.3 to <0.5 = moderate correlation, ≥0.5 to <0.7 = high correlation, and ≥0.7 to 1.0 = very high correlation [20]. Sensitivity analysis was conducted using Spearman correlations.

The ability of RAPID3 and RADAI to detect changes in patient-reported disease activity from baseline to week 12 in CZP patients was quantified as effect size (ES = mean change/standard deviation [SD] of baseline score) and relative efficiency (RE = ES^2^ of parameter *x*/ES^2^ of reference parameter) [20], with DAS28[CRP] used as the reference measure.

Number needed to treat (NNT) was assessed using the following formula: NNT = 1/(response in active treatment − response in placebo group) [20]. Missing data for parameters on which NNT was calculated or MCID was investigated were imputed using non-responder imputation.

## Results

### Patients

A total of 1063 patients were randomized, of whom 212 were entered in the placebo arm and 851 in the CZP arm. Of the 1063 randomized patients, 955 (89.8 %) completed the 12-week, double-blind phase: 184 (86.8 %) in the placebo group and 771 (90.6 %) in the CZP group. All 955 patients were entered in the open-label extension, and 809 (84.7 %) completed week 28. A similar percentage of patients in both the placebo (*n* = 80, 37.7 %) and CZP groups (*n* = 320, 37.6 %) had prior anti-TNF exposure at RCT baseline. Details of the REALISTIC trial, including primary and secondary outcomes, were previously reported [10]. At baseline, the patients’ mean age was 55 years, 78 % were female, and mean disease duration was 8.7 years. The patients had active RA with, on average, high DAS28(CRP) and high PRO scores at baseline (Table 1). The REALISTIC study met its primary efficacy endpoint (a statistically significant higher CZP ACR 20 % response rate at week 12 compared with placebo) [10].

**Table 1:** Baseline disease characteristics and PRO scores in the ITT population

	Placebo (*n* = 212)	CZP^a^ (*n* = 851)
Clinical characteristics		
Disease duration, yr, median (min–max)	6.3 (0.3–49.0)	5.43 (0.2–52.0)
Tender joint count, mean (SD)^b^	14.7 (6.6)	14.7 (6.6)
Swollen joint count, mean (SD)^b^	11.1 (5.2)	11.8 (5.6)
DAS28(CRP), mean (SD)	5.7 (0.9)	5.7 (0.9)
DAS28(ESR), mean (SD)	6.4 (0.9)	6.4 (0.9)
Baseline patient-reported outcomes		
Fatigue Assessment Scale score, mean (SD) (0––10)	6.4 (2.2)	6.2 (2.2)
Medical Outcomes Study Sleep Problem Index II score, mean (SD) (0–100)	48.1 (19.9)	47.6 (19.5)
Pain VAS score, mean (SD) (0–100)	62.3 (22.9)	58.8 (23.3)
PtGA VAS score, mean (SD) (0–100)	61.6 (20.7)	59.2 (22.1)
RADAI-TS, mean (SD) (0–10)	5.7 (1.9)	5.6 (1.8)
RAPID3, mean (SD) (0–30)	15.5 (5.4)	14.7 (5.5)

*CRP* C-reactive protein, *CZP* certolizumab pegol, *DAS28* Disease Activity Score in 28 joints, *ESR* erythrocyte sedimentation rate, *PRO* patient-reported outcome, *SD* standard deviation, *VAS* visual analog scale, *FAS* Fatigue Assessment Scale, *PtGA* Patient Global Assessment of Disease Activity, *ITT* intention to treat, *RADAI-TS* Rheumatoid Arthritis Disease Activity Index total score, *RAPID3* Routine Assessment of Patient Index Data 3

^a^CZP dose: 400 mg at weeks 0, 2, and 4 (loading dose), then 200 mg at weeks 6, 8, and 10

^b^28-joint assessment

### Impact of CZP on PROs of fatigue, sleep, pain, PtGA, HAQ-DI, RADAI-TS, and RAPID3

**Fig. 1:**
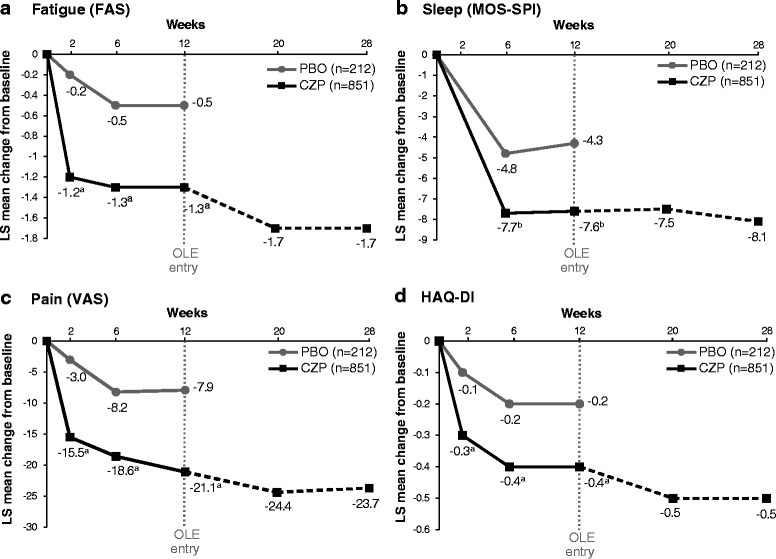
Adjusted mean least squares (LS) change from baseline in **a** fatigue, **b** sleep disturbance, **c** pain, and **d** Health Assessment Questionnaire-Disability Index (HAQ-DI) (intention-to-treat population, last observation carried forward; placebo *n* = 212, CZP *n* = 851). ^a^ *p* < 0.001 for CZP vs. placebo by analysis of covariance (ANCOVA); ^b^ *p* ≤ 0.01 for CZP vs. placebo by ANCOVA. *CZP* certolizumab pegol; *FAS* Fatigue Assessment Scale, *MOS-SPI* Sleep Problem Index II domain of the 12-item Medical Outcomes Study Sleep Scale, *OLE* open-label extension, *VAS* visual analogue scale

As shown in Fig. 1, early and clinically meaningful improvements in PROs were observed. Improvements in HAQ-DI, pain, and fatigue were reported with CZP compared with placebo from week 2 (first time point assessed) through week 12 (end of RCT) (Fig. 1a, c, d). Sleep disturbance was significantly reduced in the CZP group from week 6 onward (Fig. 1b). Improvements observed from baseline to week 12 were maintained to week 28 in the CZP-treated patients (Fig. 1). Clinically important improvements in PtGA were also observed in CZP-treated patients compared with placebo-treated patients from week 2 (PtGA least squares mean CFB −2.6 placebo vs. −14.9 CZP; *p* < 0.001) to week 12 (PtGA CFB −7.7 placebo vs. −20.4 CZP; *p* < 0.001). Improvements were maintained up to week 28 in the CZP-treated patients (PtGA CFB −24.3 at week 28).

At week 12, compared with placebo patients, more CZP patients had improvements greater than or equal to the MCID for fatigue (56.4 % vs. 46.2 %; *p* = 0.008), sleep problems (49.7 % vs. 42.5 %; *p* = 0.058), pain (59.0 % vs. 42.0 %; *p* < 0.001), and PtGA (59.5 % vs. 42.5 %; *p* < 0.001). Differences between treatment arms for MCID improvement were seen by week 2 for fatigue (*p* < 0.001), pain (*p* < 0.001), and PtGA (*p* < 0.001). Rates of CZP patients reporting MCID were maintained up to week 28 (fatigue 64.4 %, sleep problems 56.2 %, pain 68.6 %, and PtGA 69.7 % [observed case]).

**Fig. 2:**
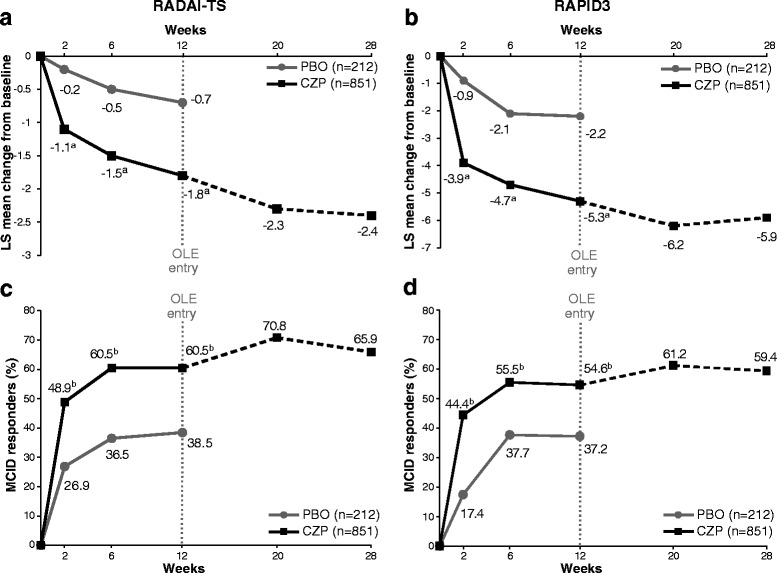
Rheumatoid Arthritis Disease Activity Index total score (RADAI-TS) and Routine Assessment of Patient Index Data 3 (RAPID3) responses. **a** Adjusted mean least squares (LS) change from baseline in RADAI-TS. **b** Adjusted mean LS change from baseline in RAPID3. **c** Percentage of patients reporting improvements greater than or equal to the minimal clinically important difference (MCID) in RADAI. **d** Percentage of patients reporting improvements greater than or equal to the MCID in RAPID3 (intention-to-treat population, placebo *n* = 212, CZP *n* = 851). ^a^ *p* < 0.001 CZP vs. placebo by analysis of covariance; ^b^ *p* < 0.001 CZP vs. placebo by logistic regression. *CZP* certolizumab pegol, *OLE* open-label extension, *PBO* placebo

Improvements in RADAI-TS and RAPID3 were observed in the CZP group compared with placebo from week 2 to week 12 (*p* < 0.001 CZP vs. placebo, all time points) (Fig. 2a, b). More CZP patients than placebo patients achieved improvements greater than or equal to the MCID in RADAI-TS and RAPID3 from week 2 to week 12 (*p* < 0.001 CZP vs. placebo, all time points) (Fig. 2c, d), with improvements maintained up to week 28 in CZP patients (Fig. 2). The NNT to achieve an MCID in RADAI-TS and RAPID3 at week 12 was approximately 4.5 and 5.6 patients, respectively. More CZP patients achieved RAPID3 scores ≤6 from week 2 onward, with 34.5 % of CZP patients and 13.7 % of placebo patients achieving RAPID3 scores ≤6 at week 12 and 40.3 % of CZP patients achieving RAPID3 scores ≤6 at week 28.

### Correlation between PROs and clinical measures of RA signs and symptoms

**Fig. 3:**
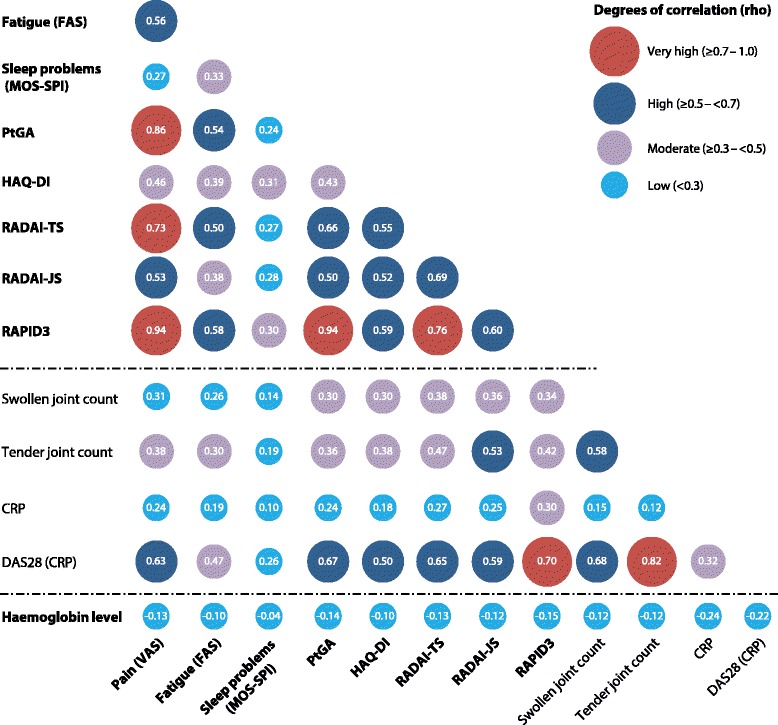
Correlations between clinical measures of rheumatoid arthritis (RA) signs and symptoms and patient-reported outcomes in overall RA population (placebo and CZP combined, intention-to-treat population). *CRP* C-reactive protein, *CZP* certolizumab pegol, *DAS28* Disease Activity Score in 28 joints, *ESR* erythrocyte sedimentation rate, *FAS* Fatigue Assessment Scale, *HAQ-DI* Health Assessment Questionnaire-Disability Index, *JS* joint score, *MOS-SPI* Sleep Problem Index II domain of the 12-item Medical Outcomes Study Sleep Scale, *PtGA* Patient Global Assessment of Disease Activity, *RADAI* Rheumatoid Arthritis Disease Activity Index, *RAPID3* Routine Assessment of Patient Index Data 3, *TS* total score

Correlations between PROs assessed at week 12 after treatment were variable (Fig. 3). Correlations greater than 0.7 were seen between patient-assessed pain and RAPID3, PtGA, and RADAI-TS and also between RAPID3 and both PtGA and RADAI-TS. Correlations between 0.5 and 0.7 were found between RADAI-JS and pain, PtGA, HAQ-DI, RAPID3, and RADAI-TS; between RADAI-TS and fatigue, PtGA, and HAQ-DI; between fatigue and pain, RAPID3, and PtGA; and between HAQ-DI and RAPID3. Other correlations were less than 0.5 (Fig. 3).

Selected PROs were correlated with several clinical indices (Fig. 3). DAS28(CRP) was correlated at 0.7 with RAPID3 and greater than 0.5 with pain, PtGA, HAQ-DI, RADAI-TS, and RADAI-JS. A correlation of 0.53 was observed between RADAI-JS and TJC. Lower correlations (i.e., less than 0.5) were observed between SJC/TJC and PROs, and correlations were generally less than 0.3 between both CRP and hemoglobin levels and PROs (Fig. 3).

Correlations were also assessed between various clinical indices of RA signs and symptoms (Fig. 3). Within this analysis, a strong correlation of 0.83 was seen between DAS28(CRP) and TJC, as well as correlations of 0.58 between SJC and TJC and 0.68 between SJC and DAS28(CRP). Correlations less than 0.5 were observed between CRP and SJC, TJC, and DAS28(CRP).

Sensitivity analyses using Spearman correlations yielded results similar to those in the primary analysis.

### Comparative responsiveness of RADAI-TS, RADAI-JS, and RAPID3 in CZP-treated patients

Effect sizes of RADAI-TS, RADAI-JS and RAPID3 between baseline and Week 12 were satisfactory in the CZP-treated patients (Table 2). Effect sizes are reported as negative since lower scores in these outcomes signify improvement in symptoms. Relative efficiency compared with DAS28(CRP) was good only in patients with over 9 affected joints at baseline (Table 2; CZP-treated patients).

**Table 2:** RAPID3, RADAI-TS, and RADAI-JS effect sizes and relative efficiency between baseline and week 12 in CZP-treated patients

	Effect size at week 12	Relative efficiency^a^ vs. DAS28(CRP) at week 12
RAPID3	−1.02	0.27
RADAI-TS	−1.10	0.32
RADAI-JS	−0.78	0.16
Baseline RADAI-JS^b^		
0–4 joints	–	0.11
5–8 joints	–	0.71
9–12 joints	–	1.60
13–16 joints	–	1.75

*CRP* C-reactive protein, *CZP* certolizumab pegol, *DAS28* Disease Activity Score in 28 joints, *RADAI* Rheumatoid Arthritis Disease Activity Index, *RAPID3* Routine Assessment of Patient Index Data 3, *JS* joint score, *TS* total score

^a^Value of 1 indicates equivalent efficiency

^b^By number of affected joints at baseline

## Discussion

We report rapid and consistent improvements in multiple PROs after CZP treatment in a group of patients with active RA. The study included patients not usually enrolled in RA clinical trials due to comorbidities, excluding only those with a history of chronic, serious, or life-threatening infection; patients with any current infection; patients with uncontrolled renal, hepatic, cardiac, or neurological disease; and patients with either concurrent or a history of malignancy. Additionally, patients with either early or late disease and a history of previous DMARD use (including prior anti-TNF, used in 40 % of patients) were permitted to participate in the study [10]. A range of correlations between PROs and clinical indices of RA signs and symptoms were seen, highlighting the importance of PROs for assessing the full impact of RA on patients. Finally, the responsiveness, as measured by ES and RE, of RADAI and RAPID3 compared with other composite clinical indices of CZP therapeutic efficacy suggests that these PROs can be used to assess treatment outcomes in trial settings.

The treatment effects of CZP on the PROs assessed demonstrated rapid onset, with clinically important improvements in pain, fatigue, and PtGA observed from the first assessment (week 2). Beneficial effects were also seen in the previously reported clinical observations of CZP therapy in the REALISTIC trial [10]. Moreover, these observations are consistent with findings from the RAPID and FAST4WARD studies of CZP in patients with RA, in which clinical benefits were seen as early as week 1 [21–23].

The ability to evaluate the clinical significance of observed improvement in parameters important to the patient is fundamental to the interpretation of clinical trials. We investigated this by analyzing whether the proportion of patients reporting MCIDs in PROs could differentiate CZP from placebo, even in a short-term trial. This approach has been validated previously using the internal anchor-based analysis to derive MCIDs. For example, PtGA and pain have been used to differentiate abatacept and placebo in patients with RA in two major RCTs [14, 24, 25]. Those studies demonstrated that MCIDs for activity limitation, fatigue, and sleep problems can be used as benchmarks in clinical trials to assess patient improvement. The results presented in the present report broadly support this conclusion. Although there was a placebo response for patients meeting MCID for PROs, consistent with observations in other randomized trials assessing PROs [26], more patients receiving active treatment achieved the MCID. Our results are concordant with the magnitude of change in PROs reported previously after CZP treatment in the RAPID trials [23, 27], as well as for other anti-TNFs in randomized and controlled studies [28]. Improvements in PRO MCID achievement after CZP therapy, compared with control patients participating in the RAPID 1 and RAPID 2 trials, were also linked to improvements in social functioning, as measured by participation in family, social, and leisure activities and productivity at work and at home [29].

We observed marked and rapid improvements from baseline in fatigue in patients who received CZP compared with patients receiving placebo, and these improvements were maintained to week 28. Fatigue is now recognized as an outcome of major importance, being highly prevalent (present in up to 70 % of patients with RA), as severe and frequent as pain, and consistently prioritized by patients themselves as one of their top outcome priorities [30]. Assessment of this PRO is strongly recommended in clinical trials of RA treatments [31]. Furthermore, fatigue is common across all rheumatic diseases, correlates with all measures of distress, and predicts dysfunction at work and overall health status [32].

Sleep disturbance is increased in patients with RA, and a prior study of MOS sleep scales in 8676 patients with RA suggested that sleep problems are linked to pain, mood, and RA disease activity [33]. In the present study, sleep improvement was associated with an improvement in fatigue measures. CZP treatment was associated with significant sleep improvement compared with the placebo treatment. TNF-α has been implicated in regulating slow-wave brain function and sleep propensity; consequently, it is thought to play a role in normal sleep cycles as well as the disturbed sleep associated with a number of disease pathologies, including RA [34, 35]. However, improvement in sleep could be due to improvement in RA signs (inflammation) and symptoms (pain). Other studies have also shown a beneficial effect of anti-TNFs on sleep in patients with RA; however, further research is required [36].

Using correlation analysis, we highlighted the value of using PROs to evaluate treatment benefit in patients with RA. Correlations between 0.3 and 0.7 were observed between clinical indices of RA signs and symptoms and the PROs PtGA, pain, and fatigue. We also found correlations between 0.3 and 0.7 for all PROs measured and disability detected by the HAQ-DI. This demonstrated that PROs measure aspects that are related to, but not the same as, clinical outcomes. However, it should be noted that sleep problems correlated poorly with pain and PtGA and also with clinical indices of RA signs and symptoms (e.g., DAS28[CRP], CRP, physician-reported TJC and SJC). This is not surprising, as sleep problems are likely multifactorial, whereas pain may be more closely related to RA disease activity, although it may still reflect damage and other comorbidities.

The results reported herein suggest that PRO indices may be useful clinical tools, complementary to other measures, for assessing RA disease activity. We found that correlations between DAS28(CRP) and RADAI or RAPID3 were between 0.5 and 0.7 (Fig. 3) in the overall RA population, and the effect sizes of these PROs were clinically important. This suggests that these PROs in particular may provide important additional information concerning treatment efficacy. Comparable responsiveness of RADAI and RAPID3 and the clinical measure DAS28(CRP) to CZP treatment was also demonstrated in patients with very active disease, further supporting this conclusion in these patients.

The NNT to achieve MCID in RADAI-TS and RAPID3 were 4.5 and 5.6 patients, respectively. While these values appear higher than those determined in the CZP RAPID trial, where NNT was 2–3 [27], this may be due to the broader patient population represented in the REALISTIC trial, combined with a shorter trial duration.

The number of “real-world” patients with RA in this study was large (*N* = 1063), especially those randomized to CZP (*n* = 851), which is a strength of this study. A possible limitation is the short-term length of the trial, which allowed for detection of early rapid improvements in PROs but excluded analysis of longer-term outcomes. In particular, 12 weeks may have been an insufficient period within which to adequately investigate the association of sleep improvements with other outcomes. HAQ-DI scores have been shown to be predictive of long-term patient prognosis [37]. Currently, there is limited information available on the predictive value of other PROs on long-term patient outcomes such as disability, mortality, and health resource use. Without this validation, PROs remain indictors of disease symptom severity rather than indicators of long-term patient prognosis. We also noted high rates of improvement greater than or equal to the MCID for PROs in patients randomized to the placebo group. As patients maintained their current treatment regimens, many patients in the placebo group were concurrently receiving MTX. As improvements in PROs observed for this group tended to plateau by week 12, the observed response may have been a consequence of patient expectations of treatment and/or regression to the mean, an effect extensively reported elsewhere [38]. We were also unable to relate PROs to radiographic changes, as radiographs were not evaluated in the REALISTIC study. It should also be noted that all reported *p* values and confidence intervals are nominal and can be interpreted only in an exploratory manner.

## Conclusions

This study highlights the rapid changes seen in a range of PROs in a representative RA population following CZP therapy. Improvement in PROs after CZP treatment indicates that the benefits of therapy extend beyond the primary composite clinical efficacy endpoints to outcomes that are more meaningful to patients, with differentiation between active treatment and placebo. RADAI and RAPID3 demonstrated very high correlation with each other and comparative responsiveness to change, which is not surprising as they assess many of the same variables. Correlations between clinical indices of RA signs and symptoms and PROs suggest that PROs can be used in conjunction with clinical outcomes to more comprehensively assess the benefits of treatment on the outcomes that are most highly valued by patients.

## Abbreviations

ACR: American College of Rheumatology
ANCOVA: analysis of covariance
CFB: change from baseline
CRP: C-reactive protein
CZP: certolizumab pegol
DAS28: Disease Activity Score in 28 joints
DMARD: disease-modifying antirheumatic drug
ES: effect size
ESR: erythrocyte sedimentation rate
FAS: Fatigue Assessment Scale
HAQ-DI: Health Assessment Questionnaire-Disability Index
ITT: intention to treat
JS: joint score
MCID: minimal clinically important difference
MOS-SPI: Sleep Problem Index II domain of the 12-item Medical Outcomes Study Sleep Scale
MTX: methotrexate
NNT: number needed to treat
OLE: open-label extension
PBO: placebo
PRO: patient-reported outcome
PtGA: Patient Global Assessment of Disease Activity
RA: rheumatoid arthritis
RADAI: Rheumatoid Arthritis Disease Activity Index
RAPID3: Routine Assessment of Patient Index Data 3
RCT: randomized clinical trial
RE: relative efficiency
REALISTIC: RA EvALuation In Subjects receiving TNF Inhibitor Certolizumab pegol
SD: standard deviation
SJC: swollen joint count
TJC: tender joint count
TNF: tumor necrosis factor
TS: total score
VAS: visual analogue scale
